# Drugs Based on NMDAR Hypofunction Hypothesis in Schizophrenia

**DOI:** 10.3389/fnins.2021.641047

**Published:** 2021-04-12

**Authors:** Qiongqiong Wu, Jing Huang, Renrong Wu

**Affiliations:** Department of Psychiatry, National Clinical Research Center for Mental Disorders, The Second Xiangya Hospital of Central South University, Changsha, China

**Keywords:** NMDA receptor, glutamate, schizophrenia, cognitive dysfunction, negative symptoms

## Abstract

Treatments for negative symptoms and cognitive dysfunction in schizophrenia remain issues that psychiatrists around the world are trying to solve. Their mechanisms may be associated with N-methyl-D-aspartate receptors (NMDARs). The NMDAR hypofunction hypothesis for schizophrenia was brought to the fore mainly based on the clinical effects of NMDAR antagonists and anti-NMDAR encephalitis pathology. Drugs targeted at augmenting NMDAR function in the brain seem to be promising in improving negative symptoms and cognitive dysfunction in patients with schizophrenia. In this review, we list NMDAR-targeted drugs and report on related clinical studies. We then summarize their effects on negative symptoms and cognitive dysfunction and analyze the unsatisfactory outcomes of these clinical studies according to the improved glutamate hypothesis that has been revealed in animal models. We aimed to provide perspectives for scientists who sought therapeutic strategies for negative symptoms and cognitive dysfunction in schizophrenia based on the NMDAR hypofunction hypothesis.

## Introduction

Schizophrenia is a psychiatric disorder afflicting approximately 1% of the population worldwide (Perala et al., 2007). It has multiple overlapping symptom clusters including positive symptoms (i.e., delusions, hallucinations), negative symptoms (i.e., social withdrawal, anhedonia), and cognitive impairments (i.e., deficits in attention, working memory, and executive function) (van Os and Kapur, 2009). Among them, negative symptoms and cognitive dysfunction have recently attracted psychiatrists’ attention. The majority of positive symptoms have been relieved by mainstream antipsychotics (the first and second generation of antipsychotics), while most negative symptoms and cognitive dysfunction remain unaffected and may even worsen with the development of schizophrenia (Lieberman et al., 2005; Lieberman, 2007; Leucht et al., 2009).

## Mechanisms of Negative Symptoms and Cognitive Dysfunction in Schizophrenia

The general mechanisms underlying negative symptoms are associated with deficient cortical dopamine transmission in mesocortical pathways and deficient serotonergic and noradrenergic transmission (Galderisi et al., 2018). There are five negative symptom domains: (1) blunted affect (diminished emotional expression); (2) alogia (diminished quantity of words spoken and spontaneous elaboration); (3) asociality (diminished social interactions and initiative); (4) avolition (diminished initiation and persistence of goal-directed activity); (5) anhedonia (diminished ability to experience pleasure) (Galderisi et al., 2018). Different domains are associated with different mechanisms in neurobiology. For example, the avolition/apathy domain involves two mechanisms, the motivational value system and the motivational salience circuit. The dysfunctional dopamine transmission in the reward circuit, especially in areas of the basal ganglia such as the ventral striatum, may cause apathy (Bortolon et al., 2018). Other domains of negative symptoms such as blunted affect and alogia remained unclear with very few revealed mechanisms (Galderisi et al., 2018).

Cognitive dysfunction, a core feature of schizophrenia, is characterized to emerge before the first episode of psychosis (FEP) and is regarded as one of the strongest predictors of functional recovery in patients with schizophrenia (Kahn and Sommer, 2015). There are eight domains in cognition: (1) attention/vigilance, (2) cognitive control/executive function, (3) reasoning/problem solving, (4) social cognition, (5) speed of processing, (6) verbal learning, (7) visual learning, and (8) working memory. Cognition is generally regulated by the hippocampus, basal ganglia, the dorsolateral prefrontal cortex, the thalamus, and regions of the motor cortex (Robison et al., 2020). Cognitive dysfunction in schizophrenia is associated with the proactive control mechanism resulting from impaired function of the dorsolateral prefrontal cortex prefrontal cortex (DLPFC) (Guo et al., 2019). The weakened interaction between DLPFC and other brain regions, as well as the neurotransmitter system disorder, such as dopamine and glutamate, contribute to cognitive dysfunction in schizophrenia (Barch and Ceaser, 2012).

The mechanisms of both negative symptoms and cognitive dysfunction are associated with NMDARs (Kahn and Sommer, 2015). First, the animal models associated with NMDARs (NMDAR antagonists induced model, gene engineered model) exhibited negative symptoms (Lee and Zhou, 2019) and impairments in cognition (recognition memory, attention, and working memory) (Thomas et al., 2017). The NMDAR hypofunction was linked to negative symptoms *via* mismatch negativity (MMN), a negative event-related potential response to a deviant stimulus in the repetitive auditory stimuli (Thiebes et al., 2017). MMN might be a biomarker for negative symptoms in schizophrenia due to glutamatergic deficits (Thiebes et al., 2017). Negative symptoms seemed to be controlled by the D3 receptors in reward and motivation circuits, which can interact with NMDARs directly (Sokoloff and Le Foll, 2017). When it comes to cognition, NMDARs in hippocampus CA1 play a predominant role in the generation of long-term plasticity (long-term potentiation and depression), and NMDAR-dependent long-term plasticity is critical in neurochemical foundations of learning and memory (Lau and Zukin, 2007; Volianskis et al., 2015). Aberrant glutamatergic inputs in dopamine circuits (mesolimbic circuit and mesocortical circuit) may induce cognitive dysfunction in schizophrenia (Robison et al., 2020). Thus, NMDAR hypofunction is closely associated with cognitive dysfunction in schizophrenia (Henneberger et al., 2010).

## NMDAR Hypofunction and Schizophrenia

There are two types of glutamate receptors: ionotropic (iGluRs) and metabotropic (mGluRs). The iGluRs are composed of α-amino-3-hydroxy-5-methylisoxazole-4-propionate receptor (AMPARs), kainate receptors (KARs), and NMDARs (also named as GluNs) (Uno and Coyle, 2019). NMDAR is highly permeable to Ca^2+^, thus playing an important role in the excitatory synapse transmission and long-term neural structural plasticity (Cull-Candy et al., 2001; Paoletti and Neyton, 2007). NMDAR is a heterotetrameric complex composed of two GluN1 subunits, which are obligatory, and with either two GluN2 subunits or a combination of GluN2/3 subunits (Paoletti and Neyton, 2007; Hansen et al., 2018). GluN2 subunits have four variants (GluN2A-D) and GluN3 subunits have two variants (GluN3A-B). There is a glycine modulatory site (GMS) on the GluN1 subunit, which binds co-agonists glycine and D-serine (Paoletti and Neyton, 2007). The GluN2 subunit has a glutamate binding site and other allosteric modulatory sites (Paoletti and Neyton, 2007). GluN1 and GluN2 subunits both have redox modulatory sites composed of the disulfide bond of cysteine residues (Lipton et al., 2002). The activation of the NMDAR requires: (1) post-synaptic depolarization induced by the activation of the AMPA receptor, which relieves the Mg^2+^ blockade of the channel; (2) glutamate binding to the GluN2 subunit and glycine or D-serine binding to the GMS on the GluN1 subunit (Paoletti et al., 2013).

The NMDAR hypofunction hypothesis emerged when the noncompetitive antagonists of the NMDAR (phencyclidine/PCP, ketamine) were found to induce nearly all three symptom clusters of schizophrenia in healthy people (Luisada, 1978; Krystal et al., 1994). Anti-NMDAR encephalitis with autoantibodies against NMDAR also supported this hypothesis because patients with anti-NMDAR encephalitis presented schizophrenia-like symptoms (Zandi et al., 2011; Kayser and Dalmau, 2016). The abundant evidence for the NMDAR hypofunction hypothesis has been extensively reviewed (Coyle, 2012; Uno and Coyle, 2019), including genetic findings (Neale et al., 2014; Yu et al., 2018), clinical findings, neuroimaging (1H-MRS, PET/SPECT), neurophysiological findings (mismatch negativity/P300/gamma band oscillations) (Wacongne, 2016; Greenwood et al., 2018; Javitt et al., 2018), and postmortem neurochemical findings.

## NMDAR-Targeted Drugs for Negative Symptoms and Cognitive Dysfunction

Drugs based on the NMDAR hypofunction hypothesis and their effects on patients with schizophrenia are summarized in Table 1.

**TABLE 1:** The summary of clinical researches on drugs augmenting NMDAR function in schizophrenia.

	Pharmacology	Time	Sample	Method	Significant effects on symptom clusters	References
Glycine, adjunctive therapy	Initially 2 g/d, upward to 0.4 g/kg/day	8 W	14 SCZ patients	PANSS	A 17.1% reduction in negative symptoms	Javitt et al., 1994
	0.8 g/kg/d	6 W	11 treatment-resistant SCZ patients	PANSS	A 7% reduction in negative symptoms	Heresco-Levy et al., 1996
	0.8 g/kg/d	6 W	22 treatment-resistant SCZ patients	BPRS, PANSS	A 30 ± 16% reduction in negative symptom, reduction in extrapyramidal Symptoms.	Heresco-Levy et al., 1999
	60 g/d	8 W	20 SCZ patients	PANSS	No significant outcome	Evins et al., 2000
	0.8 g/kg/d	6 W	12 SCZ patients	PANSS	A 34% reduction in Negative symptoms	Javitt et al., 2001
	0.8 g/kg/d	1 W	16 healthy participants	MMN	Reduction in duration MMN amplitude	Leung et al., 2008
	Acute administration: 0.2 g/kg; chronic treatment: incremented to 0.6 g/kg/d	6 W	22 SCZ patients	MMN, PANSS	Acute glycine administration increased duration MMN; chronic glycine administration improved negative symptoms	Greenwood et al., 2018
GlyT1 inhibitor- sarcosine, adjunctive therapy	2 g/d sarcosine	6 W	65 SCZ patients	PANSS,SANS	All three symptom clusters (≥30% reduction in the PANSS total score)	Lane et al., 2005
	2 g or 1 g/d sarcosine	6 W	20 acutely symptomatic drug-free SCZ patients	PANSS	A ≥20% reduction in the total scores in PANSS	Lane et al., 2008
	Sarcosine (in the first stage 5 patients received 2 gm/d of sarcosine for 1 week and in the second stage 17 patients received 4 gm/d for one week)	8 D	22 SCZ patients	PANSS, CGI, MCCB	Improvements in positive symptoms, negative symptoms, speed of processing	Amiaz et al., 2015
GlyT1 inhibitor- bitopertin, monotherapy	Bitopertin (10, 30, or 60 mg/d)	8 W	phase 2 proof-of-concept trial involved 323 SCZ patients with predominant negative symptoms	PANSS	Negative symptoms improved in the 10, 30 mg/d group (response rate = 65% in the 10 mg/d)	Umbricht et al., 2014
	Bitopertin 10, 30 mg/d alone	4 W	301 SCZ patients	PANSS	No significant Outcome	Bugarski-Kirola et al., 2014
GlyT1 inhibitor- org 25935, adjunctive therapy	Org 25935 (4 to 8 mg twice daily and 12 to 16 mg twice daily)	12 W	187 SCZ patients	SANS the Scale for Assessment of Negative Symptoms 1-22	No significant Outcome	Schoemaker et al., 2014
GlyT1 inhibitor- AMG747, adjunctive therapy	AMG747 orally receive daily AMG 747 (5 mg, 15 mg, or 40 mg)	12 W	153 SCZ patients	Negative Symptom Assessment (NSA)-16 total score	No significant outcome	Dunayevich et al., 2017
D-serine, monotherapy	D-serine (Week 1: 1.5 g/d Week 2-10: 3 g/d) versus olanzapine (Week 1: 15 mg/d Week 2-10: 30 mg/d)	10 W	18 SCZ treatment-resistant patients	PANSS	Compared to olanzapine, D-serine has less improvement in PANSS total scores	Ermilov et al., 2013
	60 mg/kg/d D-serine	16 W	35 participants at clinical high risk of schizophrenia	SOPS	A 35.7% reduction in prodromal symptoms at high-risk group	Kantrowitz et al., 2015
	60 mg/kg/d D-serine	6 W	16 SCZ patients	MMN	Improvement in MMN	Kantrowitz et al., 2018
D-serine, adjunctive therapy	30 mg/kg/d	6 W	31 SCZ patients	Wisconsin Card Sorting Test	Improvement in all three symptom clusters; a 17% reduction in positive symptoms; a 21% reduction in negative symptoms; 12% improvements in cognition	Tsai et al., 1998
	30 mg/kg/d, added to clozapine	6 W	20 SCZ patients	CGI, PANSS	No significant Outcome	Tsai et al., 1999
	30 mg/kg/d D-serine	6 W	39 SCZ patients	PANSS	Improvement in three symptom clusters; 39% showed a >20% improvement in total BPRS score	Heresco-Levy et al., 2005
	30, 60, or 120 mg/kg/day D-serine	4 W	42 SCZ patients	PANSS, MATRICS	Improvement in all three symptom clusters	Kantrowitz et al., 2010
	2 g/d	16 W	195 SCZ patients	SANS, MATRICS	No significant Outcome	Weiser et al., 2012
D-cycloserine, adjunctive therapy	5, 15, 50, and 250 mg/d DCS	2 W	9 SCZ patients	SANS	A 21% improvement in negative symptoms	Goff et al., 1995
	50 mg/d DCS, clozapine	13 W	17 SCZ patients	PANSS, SANS, GAS	Worsened negative symptoms	Goff et al., 1999
	50 mg/d DCS, risperidone	2 W	10 SCZ patients	SANS	A 10% improvement in negative symptoms	Evins et al., 2002
	50 mg/d	4 W	22 SCZ patients	SANS	No significant Outcome	Rothbaum et al., 2014
	50 mg/W	8 W	33 SCZ patients	The Logical Memory Test, SANS	A 16.6% net reduction in negative symptoms	Goff et al., 2008
	50 mg/W	8 W	36 SCZ patients	The auditory discrimination task, SANS, MATRICS	Improvement in long-term memory (LTM) on the practiced auditory discrimination task; 26% reduction in SANS scores	Cain et al., 2014
	50 mg/d	6 W	41 SCZ patients	PANSS, SANS	No significant outcome in three symptoms	Takiguchi et al., 2017
DCS, monotherapy	100 mg	3 h	45 SCZ patients	EEG paradigm	Cognition (working memory, experience-dependent neuroplasticity)	Forsyth et al., 2017
	100 mg		32 healthy participants	LTP EEG paradigm, cognitive task**s**	Cognition (experience-dependent neuroplasticity)	Forsyth et al., 2015
DAAO inhibitor- sodium benzoate, adjunctive therapy	1 g/d sodium benzoate	6 W	52 SCZ patients	PANSS	All three symptom clusters. (a 21% reduction of PANSS scores)	Lane et al., 2013
	1 g/d, 2 g/d sodium benzoate, added to Clozapine	6 W	60 clozapine-resistant SCZ	PANSS, SANS, GAF,	Small improvements in overall symptomatology	Lin et al., 2018
Antioxidant						
NAC, monotherapy	2.4 g/d NAC	1 W	20 SCZ patients	MRI	NAC can reduce medial frontal resting-state functional connectivity (rs-FC)	McQueen et al., 2019
NAC, adjunctive therapy	1 g/d	24W	140 chronic SCZ patients	PANSS, CGI	Moderate benefits Negative symptoms	Berk et al., 2008
	2 g/d	16W	11 SCZ patients	MMN	Improvement in MMN generation	Lavoie et al., 2008
	2 g/d	crossover design, 16 W	11 SCZ patients	resting-state EEGs	NAC modulates EEG synchronization	Carmeli et al., 2012
	NAC (up to 2 g/d), risperidone (up to 6 mg/d)	8 W	42 SCZ chronic patients with predominant negative symptoms	PANSS negative subscale	Negative symptoms	Farokhnia et al., 2013
	0.6 g/d NAC, risperidone (up to 4-6 m g / d)	8 W	121 SCZ first-phase patients	PANSS, weight, lipid metabolism	Positive and negative symptoms, lipid metabolism, and weight control	Zhang et al., 2015
	3.6 g/d	52 W	60 early phase schizophrenia patient	PANSS, CGI, PSP, BACS, MRI	Negative symptoms	Breier et al., 2018
	2.7 g/d	72W	15 early psychosis patients	low-level auditory processing	Cognitive dysfunction (low-level auditory processing)	Retsa et al., 2018
	2.7 g/d	72W	63 early psychosis patients	PANSS, neurocognition, and redox markers	Neurocognition (processing speed) and brain redox state.	Conus et al., 2018
	1.2 g/d	12 W	84 SCZ patients	PANSS	All three symptom clusters: cognition (speed of processing and attention, working memory)	Sepehrmanesh et al., 2018
	1.2^_^2.4 g/d NAC, clozapine	4 W	5 SCZ patients developed sialorrhea during clozapine treatment (300–450 mg/day)	the Visual Analog Scale	Clozapine-induced sialorrhea	Uzun et al., 2019
Sulforaphane, adjunctive therapy	30 mg/d sulforaphane-glucosinolate	8 W	10 SCZ patients	PANSS	Cognitive dysfunction	Shiina et al., 2015
	100 μmol sulforaphane	1 W	9 healthy participants	MRS	Improvement in brain GSH levels and redox state.	Sedlak et al., 2018

*SCZ, schizophrenia/schizophrenic; GlyT1, Glycine transporter-1; DCS, D-cycloserine; DAAO, D-amino acid oxidase; NAC, N-acetylcysteine; SANS, the Scale for the Assessment of Negative Symptoms; MATRICS, the Measurement and Treatment Research to Improve Cognition; SOPS, the Scale of Prodromal Symptoms; NAS, the Negative Symptom Assessment; BPRS, the Brief Psychiatric Rating Scale; CGI, the Clinical Global Impression Severity and Improvement scales; PSP, the Personal and Social Performance Scale; GAS, Global Assessment Scale; GAF, the Global Assessment of Functioning; BACS, the Brief Assessment of Cognition in Schizophrenia.*

### Targeting the Glycine Modulatory Site (GMS)

**FIGURE 1:**
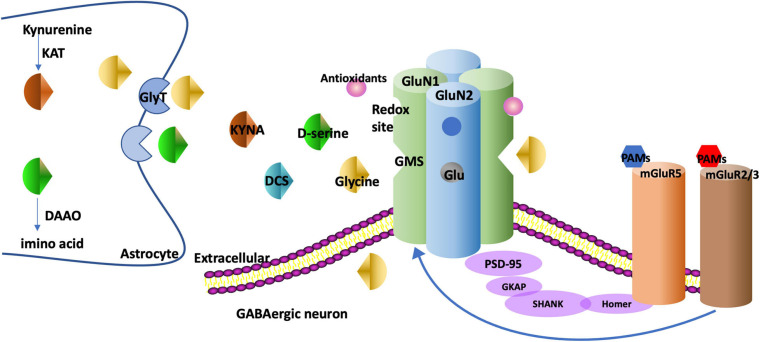
The targets of drugs augmenting NMDAR function. NMDAR is generally composed of two GluN1 and two GluN2 subunits. There is a GMS in GluN1. Glycine and d-serine bind to the GMS as co-agonists. DCS binds to the GMS as a partial agonist and KYNA binds to the GMS as a competitive antagonist. GlyT-1, the glycine transporter type, is expressed at glutamatergic synapses to modulate glycine uptake. DAAO is an intracellular enzyme degrading d-serine into amino acids. KAT regulates the production of KYNA in astrocytes. The metabotropic glutamate receptors such as mGluR5s and mGluR2/3s have multiple interactions with NMDARs. For example, mGluR5s and NMDARs are linked physically *via* scaffolding proteins Homer, SHANK. The PAMs of these receptors regulate metabotropic glutamate receptors and NMDARs as well. NMDARs have redox sites which can be regulated by antioxidants. GMS, glycine modulatory site; DCS, D-cycloserine; KYNA, Kynurenic acid; GlyT, glycine transporter; DAAO, D-amino acid oxidase; KAT, Kynurenine aminotransferase; PAM, positive allosteric modulator; PANSS, the Positive and Negative Syndrome Scale; SANS, Scale for the Assessment of Negative Symptoms.

Glycine, d-serine, D-cycloserine (DCS), and kynurenic acid (KYNA) can bind to the GMS on the GluN1 subunit. Glycine and d-serine function as co-agonists, and DCS is a partial agonist while KYNA is a competitive antagonist. The GMS is not saturated in brains and thus the NMDAR function can be augmented *via* stimulating the GMS (Balu and Coyle, 2015). Drugs targeting GMS on GluN1 have been discovered, as is shown in Figure 1. On the contrary, glutamate as an agonist for GluN2 has a tendency to induce excitotoxicity and epilepsy (Uno and Coyle, 2019), thus few drugs targeting GluN2 have been discovered (Uno and Coyle, 2019).

#### Glycine and Glycine Transporter-1 (GlyT-1) Inhibitors

Glycine has been administrated with mainstream antipsychotics since 1994. The neural function has an inverted-U relationship with the synaptic glycine concentration (Greenwood et al., 2018), which is associated with the internalization of NMDARs from the membrane to the cytoplasm (Nong et al., 2003). The efficacy of glycine in improving negative symptoms has been confirmed in schizophrenia (Javitt et al., 1994, 2001; Heresco-Levy et al., 1996, 1999). A significant improvement (around 30% reduction rates) in negative symptoms has been revealed by comparing the PANSS scores after glycine administration (Heresco-Levy et al., 1996; Javitt et al., 2001). Furthermore, glycine is effective for negative symptoms in treatment-resistant schizophrenic patients (Heresco-Levy et al., 1996, 1999). In addition, acute glycine administration improved MMN both in healthy controls and patients with schizophrenia (Leung et al., 2008; Greenwood et al., 2018). However, some studies reported no significant improvements in negative symptoms or cognitive dysfunction (Evins et al., 2000; Buchanan et al., 2007). Glycine has some disadvantages. It requires a high dose (0.8 g/kg/day) to have an effect, and it may induce long-term depression (Greenwood et al., 2018) and digestive dysfunction (Heresco-Levy et al., 1999).

Glycine transporter-1 is a predominant modulator for the intra/extra-cellular glycine concentration *via* regulating glycine reuptake (Hashimoto, 2010). GlyT-1, a subtype of GlyT, co-localized with NMDAR and is primarily expressed in the synapse of glutamatergic neurons (Hashimoto, 2011). When GlyT-1 is inhibited, the extra-cellular glycine concentration rises, and subsequently the NMDAR function is augmented (Hashimoto, 2010). Thus, GlyT-1 inhibitors (such as sarcosine, bitopertin, org 25935, AMG747) are promising for improving negative symptoms and cognitive dysfunction in schizophrenia. They are classified into competitive antagonists (sarcosine) and noncompetitive antagonists (bitopertin, org 25935, AMG747) (Hashimoto, 2010).

Bitopertin/RG1678, as the first potent GlyT-1 inhibitor, has reported marked effects on negative symptoms in preclinical studies (Pinard et al., 2018). However, the third phase of the clinical trial of bitopertin (Bugarski-Kirola et al., 2014; Umbricht et al., 2014; Pinard et al., 2018) failed, and another two noncompetitive GlyT-1 inhibitors org 25935 (Schoemaker et al., 2014), AMG747 (Dunayevich et al., 2017) failed to have positive results in their phase II trials as well. It was suspected that the modest effect of GlyT1 inhibitors may be associated with the preferential action of glycine on extra-synaptic NMDAR function rather than synaptic NMDAR function (Papouin et al., 2012). However, there are still some GlyT-1 inhibitors such as PF-03463275 (D’Souza et al., 2018) and BI-425809 (Moschetti et al., 2018) in phase II trials and some have exhibited the potential to improve memorization and cognitive dysfunction in the preclinical phase (Harada et al., 2012; D’Souza et al., 2018).

Sarcosine (N-methylglycine), an endogenous competitive GlyT-1 antagonist, is generated as an intermediate in glycine synthesis and degeneration (Lane et al., 2008). After its pharmacokinetic evaluations and safety was confirmed in humans (Amiaz et al., 2015), sarcosine exhibited improvements in positive symptoms, negative symptoms, and general psychopathology (Amiaz et al., 2015). The cognitive dysfunction (especially speed of processing), was significantly improved after sarcosine administration in patients with schizophrenia (Amiaz et al., 2015). In addition, sarcosine was found to be beneficial in both the chronic (Lane et al., 2010; Amiaz et al., 2015) and acute phase of schizophrenia (Lane et al., 2005, 2008).

#### D-Serine, DCS, D-Amino Acid Oxidase (DAAO) Inhibitors

D-serine is localized especially in GABAergic neurons and astrocytes (Wolosker et al., 2008). It is generated by the serine racemases (SRs) in neurons and degenerated by DAAOs or SRs in astrocytes. Compared with glycine, D-serine presented closer associations with schizophrenia and induced a more powerful activation of synaptic NMDARs because D-serine can serve as a monotherapy in schizophrenia (MacKay et al., 2019). The first study that added D-serine with the non-clozapine mainstream antipsychotics revealed that D-serine improved all three symptom clusters in chronic treatment-refractory schizophrenia (Tsai et al., 1998). However, whether the mainstream antipsychotic is clozapine or not can affect the efficacy of adjunctive D-serine. When follow-up studies administered D-serine with clozapine, the results were negative (Tsai et al., 1999). In the studies that followed, some found that adjunctive D-serine was effective in all three symptom clusters (Heresco-Levy et al., 2005; Kantrowitz et al., 2010) while some found it to be ineffective (Lane et al., 2005; Weiser et al., 2012). These negative results may be due to the narrow therapeutic window of D-serine. A high dose (60 mg/kg/day) of D-serine is required to achieve adequate brain concentration and potentiate NMDAR activation (MacKay et al., 2019), because its oral bioavailability is low (Iwata et al., 2015). However, a high dose of D-serine can lead to nephrotoxicity (Iwata et al., 2015). These features of D-serine limit its application in patients with schizophrenia. Owing to its possible effects on positive symptoms, the effects of D-serine monotherapy were explored in patients with schizophrenia (Tsai et al., 1998). The studies that followed compared the efficacy of D-serine monotherapy and high-dose olanzapine monotherapy, because olanzapine is recognized as a recommended medication for schizophrenia (Ermilov et al., 2013). However, the D-serine has less reduction in PANSS total scores compared to high-dose olanzapine (Ermilov et al., 2013). Of note, D-serine can improve prodromal symptoms in participants who are at a clinically high risk of schizophrenia (Kantrowitz et al., 2015). In addition, a recent study reported an improvement in MMN in patients with schizophrenia when treated with D-serine for just 1 week (Kantrowitz et al., 2018).

D-amino acid oxidase is a catalyzing flavoprotein in the oxidative deamination of neutral D-amino acids, and the inhibition of DAAOs can augment NMDAR function as a treatment for schizophrenia. Multiple DAAO inhibitors were discovered with wide structural variety (Szilagyi et al., 2018). The first-generation of DAAO inhibitors is carboxylic acids. Sodium benzoate, a food preservative with good safety, is a DAAO inhibitor of the first generation with moderate activation (Lin et al., 2018). When sodium benzoate was administrated with risperidone or haloperidol, a wide variety of symptom domains, including neurocognition, were improved in patients (Lane et al., 2013). When added to clozapine, sodium benzoate improved three symptom clusters, and the 2 g/day dose had better results than the 1 g/day dose in clozapine-resistant schizophrenia (Lin et al., 2018). However, the D-serine level in the brain was not altered when the patients were administered sodium benzoate (Lin et al., 2018). It challenged the expected working mechanism of benzoate as a DAAO inhibitor in brain. In addition to DAAO inhibition, sodium benzoate can serve as an antioxidant, protecting neurons from oxidative stress (Lin et al., 2018) and it may affect the D-serine/L-serine cycle as well (Matsuura et al., 2015). Another clinical trial to explore the efficacy of sodium benzoate in early psychosis is ongoing (Ryan et al., 2017). In the discovery of other generations of DAAO inhibitors, bioisosteric moiety replaced the carboxylic group in second-generation inhibitors and cyclic carboxylic acid bioisosters connected to bulky nonpolar moieties with a flexible linker in third-generation inhibitors (Szilagyi et al., 2018). Other novel DAAO inhibitors, such as PGM030756 (Howley et al., 2017), are still in pre-clinical studies and need further confirmation of their efficacy in humans for species difference in D-serine metabolism (Szilagyi et al., 2018).

#### D-Cycloserine (DCS)

D-cycloserine, a partial agonist for GMS, increases the NMDAR channel open time and possibility at low doses (Dravid et al., 2010). It is noted that DCS has an inverted-U dose response curve similar to glycine, and chronic DCS administration may facilitate NMDAR internalization and become ineffective (Goff et al., 1995; Goff, 2016). A high dose of DCS functions as an antagonist of GMS competing with other full agonists like D-serine and glycine (Uno and Coyle, 2019). The benefits of adjunctive DCS in negative symptoms was reported early in 1995, and 50 mg/day was reported as the best dose with significant outcomes (Goff et al., 1995, 1996). However, the studies that followed found that when DCS was added with clozapine, the negative symptoms worsened in a 13-week follow-up (Goff et al., 1999). It is probably because clozapine differs from other antipsychotics in releasing D-serine and glutamate to enhance NMDARs, and DCS may attenuate the positive effects of clozapine *via* competing for GMS (Goff et al., 1999). When DCS was added with non-clozapine antipsychotics like risperidone, negative symptoms were improved (Evins et al., 2002). The intermittent DCS administration (per week) proved to be effective as well (Goff, 2016). It was revealed that taking DCS 50 mg per week improved cognitive tasks such as memory consolidation (Goff et al., 2008) and auditory discrimination task (Cain et al., 2014). Cognitive behavioral therapy for positive symptoms was improved if DCS was taken 1 h prior to an intervention (Gottlieb et al., 2011). Recent studies revealed the working memory and experience-dependent plasticity were enhanced in healthy participants (Forsyth et al., 2015) or patients with schizophrenia (Forsyth et al., 2017). However, some negative results have also been reported in the administration of DCS as adjunctive therapy, and a high dose (100 mg) of DSC aggravated symptoms of early-onset schizophrenia (van Berckel et al., 1999; Buchanan et al., 2007; Takiguchi et al., 2017), because a high dose can function as antagonists of GMS. Thus, the therapeutic window of DCS seems to be crucial in the application of DCS (Cascella et al., 1994). It was revealed that the effects of DCS were associated with the onset age of schizophrenia and the white matter integrity (Takiguchi et al., 2017). In conclusion, DCS features in improving negative symptoms (10-26%) and part of cognitive dysfunction (working memory, experience-dependent neuroplasticity) in patients with schizophrenia (Goff, 2017).

#### Kynurenine Aminotransferase (KAT) Inhibitors Targeting KYNA

Kynurenic acid is an endogenous competitive antagonist for GMS, and its production is catalyzed by KATs in astrocytes (Plitman et al., 2017). The association between the increased brain KYNA levels and subsequent cognitive dysfunction in schizophrenia was revealed by both human and animal studies (Bortz et al., 2017; Plitman et al., 2017). The underlying mechanisms may be the dysregulation of kynurenine metabolism which leads to dorsolateral prefrontal cortex volume loss and attention impairment in patients with schizophrenia (Kindler et al., 2020). Thus, the inhibition of KATs is supposed to enhance NMDAR function as a therapeutic target for schizophrenia (Nematollahi et al., 2016). But this strategy may involve another controversial mechanism independent from NMDARs, which is associated with nicotinic acetylcholine receptors (nAChRs): KYNA is also a non-competitive antagonist of the α7nAChR and the decreased KYNA can reverse α7nAChRs dysfunction in schizophrenia (Albuquerque and Schwarcz, 2013; Plitman et al., 2017). However, most evidence supported kynurenate acting *via* NMDARs (Stone, 2020). The potent KAT-I inhibitors include phenylhydrazone hexanoic acid derivatives and KAT-II inhibitors include a pyrazole series of compounds (Jayawickrama et al., 2015). Although KAT inhibitors (such as BFF816 Bortz et al., 2017) were reported to have good efficacy in cognitive dysfunction in animal models, this strategy has not been confirmed in humans yet.

### Positive Allosteric Modulators (PAMs) for Metabotropic Glutamate Receptors (mGluRs)

The direct therapeutic targets for NMDARs were challenged for the widespread distribution of NMDARs in the brain and the potential toxicity induced by the hyperglutamatergic condition. Thus, PAMs for mGluRs were discovered because they reversed NMDAR hypofunction but did not induce glutamatergic overexcitation (Field et al., 2011). The mGluRs have eight subtypes and they can be classified into three groups: Group I includes mGluR1 and mGluR5; Group II includes mGluR2 and mGluR3; Group III includes mGluR4, mGluR6-8 (Lundstrom et al., 2016). Nearly all of the subtypes of mGluRs have been recognized as promising therapeutic targets for schizophrenia. Among them, the mGlu5Rs and Group II mGluRs have been extensively researched, which we will discuss in the following section (Maksymetz et al., 2017; Stansley and Conn, 2018). PAMs refer to the agents binding to allosteric binding sites to potentiate the targeted receptor function (Ellaithy et al., 2015). They have apparent advantages over traditional agonists because they activate the receptors indirectly and they are less likely to cause NMDAR internalization which interferes with the effects of glycine and DCS (Field et al., 2011).

#### PAMs of mGlu5 Receptors

The mGluR5 has functional associations with NMDARs in GABAergic interneurons as they have some overlapping intracellular signaling pathways (Matosin and Newell, 2013). mGluR5s and NMDARs are linked physically *via* scaffolding proteins Homer, SHANK, guanylate-kinase-associated (GKAP), and post-synaptic density 95 (PSD95) as is shown in Figure 1 (Matosin and Newell, 2013). The mGluR5 PAMs can subsequently activate mGluR5s and NMDARs. VU0409551 (Conde-Ceide et al., 2015), a well-developed PAM, was reported to augment NMDAR function and improve cognitive dysfunction in the animal models of schizophrenia (Balu et al., 2016). mGluR5 PAMs may function *via* selectively potentiating mGluR5 coupling to Gαq-mediated signaling independent of NMDAR (Rook et al., 2015; Ghoshal et al., 2017). However, the safety and efficacy of mGluR5 PAMs have not yet been confirmed in clinical studies.

#### PAMs of Group II mGlu Receptors

Group II mGlu receptors have been shown to modulate glutamatergic activity in brain synapses (Fell et al., 2012). The activation of mGluR2/3s in the postsynaptic membrane promoted NMDAR currents *via* Src kinase (Trepanier et al., 2013), protein kinase C (Tyszkiewicz et al., 2004), or SNARE protein (Cheng et al., 2013). Furthermore, Group II agonists decreased excessive glutamate release in animal models of schizophrenia (Marek, 2010). Furthermore, mGluR2s was reported to have interaction with 5-HT_2A_ receptors, which are the targets of second generation antipsychotics as well (Ellaithy et al., 2015). A mixed mGlu2/3 receptor agonist LY2140023 failed in Phase II trials when it was administered with olanzapine (Kinon et al., 2011), even though it had shown good efficacy in positive and negative symptoms of schizophrenia in a previous study (Patil et al., 2007). Two mGlu2 PAMs have reached clinical trials: AZD8529 from AstraZeneca and JNJ-40411813/ADX71149 from Janssen Pharmaceuticals, Inc. and Addex Therapeutics (Field et al., 2011). The Phase IIa study of ADX71149 demonstrated the efficacy of adjunctive ADX71149 in improving the residual negative symptoms in patients with schizophrenia^1^. But AZD8529 failed to have a significant difference to the placebo in a phase IIa proof of concept (POC) study^2^ ^,3^.

### Antioxidants

Oxidative stress is defined as an imbalance between pro-oxidants and antioxidants. It was speculated that redox state and NMDAR activity interacted with each other in schizophrenia (Hardingham and Do, 2016) and these two individual factors both had developmental effects on parvalbumin-expressing GABAergic interneurons in schizophrenia (Korotkova et al., 2010; Hasam-Henderson et al., 2018). The underlying mechanisms are that the redox state regulates the disulfide bond formation of cysteine residues in GluN1 and GluN2A (also called the redox modulatory site) and subsequently regulates NMDAR activity (Lipton et al., 2002). Glutathione (GSH), protective from reactive oxidative stress, can bind to the redox modulatory site of NMDARs. GSH deficits and oxidative stress may induce the NMDAR hypofunction state in schizophrenia (Steullet et al., 2006). In addition, the conventional antipsychotics such as clozapine and risperidone, can induce oxidative stress and cause neural injury. Thus, the adjunctive therapy of antioxidant has the potential to reverse oxidative stress and NMDAR hypofunction in patients with schizophrenia (Hardingham and Do, 2016). Although there are various types of antioxidants that are beneficial to negative symptoms and cognitive dysfunction in schizophrenia (Magalhaes et al., 2016), few antioxidants have been reported to have an effect associated with NMDARs, except for N-acetylcysteine (NAC) and Sulforaphane.

#### N-Acetylcysteine (NAC)

N-acetylcysteine (NAC) is a precursor of L-cysteine, contributing to glutathione synthesis (Chen et al., 2016). It passes the blood-brain barrier easily and is involved in diverse pathways in the pathology of schizophrenia, such as brain GSH regulation, glutamatergic transmission, inflammatory pathways, and mitochondrial function (Minarini et al., 2017). In NMDAR hypofunction models, NAC protected mice from mitochondrial and synaptic injury (Phensy et al., 2017a). A two-hit model (the exposure to perinatal infection, peripubertal unpredictable stress) of schizophrenia confirmed the benefits of NAC as well (Monte et al., 2020). The synaptic deficits of schizophrenia can be reversed by NAC as revealed in cortical interneurons derived from human induced stem cells (Kathuria et al., 2019).

In clinical studies, NAC as adjunctive therapy was found to ameliorate severity of all three symptom clusters in patients with schizophrenia (Berk et al., 2008; Farokhnia et al., 2013; Zhang et al., 2015; Breier et al., 2018; Conus et al., 2018; Retsa et al., 2018; Sepehrmanesh et al., 2018; McQueen et al., 2019; Uzun et al., 2019). Four of these studies recruited first-phase or early phase patients with schizophrenia (Zhang et al., 2015; Breier et al., 2018; Conus et al., 2018; Retsa et al., 2018) and the others studied the effects of NAC in chronic schizophrenia (Sepehrmanesh et al., 2018). Another study on the administration of NAC for 26 weeks will be recruiting 162 young people in first-episode psychosis (Cotton et al., 2019). NAC has been added to different antipsychotics such as risperidone (Farokhnia et al., 2013) and clozapine (Uzun et al., 2019) to explore its efficacy in schizophrenia, and all the combinations reported improvements in negative symptoms and cognitive dysfunction. Of note, NAC was discovered to ameliorate clozapine-induced sialorrhea in a case series (Uzun et al., 2019). A significant improvement in lipid metabolism and weight control was noted as it was difficult for patients to maintain weight and spare them from metabolic disorders during the administration of the second-generation antipsychotics (Zhang et al., 2015). Sepehrmanesh Z found that NAC had positive effects on positive symptoms as well (Sepehrmanesh et al., 2018). A meta-analysis in 2018 included three randomized controlled trials (RCTs) on NAC in schizophrenia and confirmed the efficacy of the adjunctive NAC (Zheng et al., 2018). In addition, NAC can alter MMN and EEG synchronization (Carmeli et al., 2012), which linked NMDAR augmentation (Lavoie et al., 2008). Recently, the efficacy of NAC monotherapy on functional signatures was explored, and it reduced medial frontal resting-state functional connectivity (McQueen et al., 2019).

#### Sulforaphane

Sulforaphane is a natural compound found in the seeds and sprouts of cruciferous plants. It is protective of inflammation and oxidative stress (Fahey and Talalay, 1999) *via* binding to kelch-like ECH-associated protein 1 (KEAP1) and activating the transcription factor NF-E2-related factor 2 (Nrf2) (Keum, 2011; Cardozo et al., 2013). Sulforaphane has advantages over NAC, because NAC reduces oxidative stress mainly *via* cysteine, but cysteine cannot be fully utilized to generate GSH while sulforaphane is functioning *via* KEAP1/Nrf2 which affects GSH more efficiently (Hardingham and Do, 2016). Furthermore, the action of sulforaphane was linked to NMDAR as sulforaphane prevented the alteration of NMDAR activity in rats (Feng et al., 2017). However, there have only been two published studies on the administration of sulforaphane in human studies associated with schizophrenia (Shiina et al., 2015; Sedlak et al., 2018). Sulforaphane-rich broccoli sprout extract (30 mg/d) improved cognitive dysfunction in patients with schizophrenia (Shiina et al., 2015). The 7-day administration of sulforaphane increased brain GSH levels both in the animal model and human sample (Sedlak et al., 2018). In a PCP-induced animal model for schizophrenia, taking sulforaphane-rich broccoli sprout extracts during juvenile and adolescence rescued the cognitive dysfunction in adulthood (Shirai et al., 2015).

## Discussion

The NMDAR hypofunction hypothesis produces novel targets for therapeutic interventions for patients with schizophrenia, especially for negative symptoms and cognitive dysfunction. Therapeutic targets to augment NMDAR function include compounds targeting the GMS on GluN1, PAMs for mGlu receptors, and antioxidants. Compounds targeting the GMS on GluN1 include glycine & GlyT-1 inhibitors, D-serine & D-amino acid oxidase (DAAO) inhibitors, DCS, and KAT inhibitors.

Most of the drugs discussed above can improve some of the negative symptoms in patients with schizophrenia. We listed the net reduction rates of PANSS-negative scores/SANS scores if they were reported in Table 1. Glycine seemed to have had the strongest effects on negative symptoms, reducing about 30% of the Positive and Negative Syndrome Scale (PANSS)-negative score. In addition, other drugs such as D-serine and D-cycloserine were reported to have >20% reduction rates on PANSS -negative scores or the Scale for the Assessment of Negative Symptoms (SANS) scores, where 20% is recognized as the cutoff threshold for meaningful improvement (Heresco-Levy et al., 2005). The meta-analyses reported that the overall effect size for NMDAR-targeted adjunctive drugs was small to moderate for negative symptoms. A meta-analysis including studies on DCS and D-serine in 2013 reported a moderate effect-size improvement while a meta-analysis in Singh and Singh (2011) and a meta-analysis in Tsai and Lin (2010) both reported a small effect-size.

Compared with their effects on negative symptoms, NMDAR-targeted drugs had less improvements for cognitive dysfunction in patients with schizophrenia. The improved cognition domains after the administration of NMDAR-targeted drugs are limited to one or two. For example, DCS was recognized as a robust agent to increase neuroplasticity and to improve working memory as revealed in DCS monotherapy studies (Forsyth et al., 2015, 2017). NAC can improve more cognition domains than DCS, which includes speed of processing, attention, and working memory (Conus et al., 2018; Sepehrmanesh et al., 2018). There are four meta-analyses exploring the effects of NMDAR-targeted drugs on cognition. Only one meta-analysis in 2010 reported positive results, while the others reported non-significant results. The meta-analysis in 2010 reported that NMDAR-targeted drugs exhibited a small effect-size improvement in cognition (Tsai and Lin, 2010). A meta-analysis including D-serine, DCS, benzoate, and NAC in 2019 demonstrated that these drugs had a small but non-significant effect on overall cognitive function, but had significant positive effects in the 30-39 year old subgroup (Chang et al., 2019). These drugs presented a significant improvement in working memory among all eight domains (Chang et al., 2019). However, another two meta-analyses both reported their non-significant effects on overall cognitive function in patients with schizophrenia (Choi et al., 2013; Iwata et al., 2015).

Most of the NMDAR-targeted drug administrations were reported as safe and well-tolerated throughout the studies without significant clinical or laboratory side-effects. Sodium benzoate as a food additive and antioxidant sulforaphane extracted from food are safe for humans. However, high-dose glycine had slight side-effects on gastrointestinal functions (Heresco-Levy et al., 1999).

Altogether, the overall effects of these NMDAR-targeted drugs had little to moderate negative symptoms and cognitive dysfunction, see Table 2. The development of NMDAR-targeted drugs has resulted in many unexpected outcomes. For example, some potential new drugs such as GlyT-1 inhibitors bitopertin/RG1678 (Bugarski-Kirola et al., 2014; Umbricht et al., 2014; Pinard et al., 2018) and GlyT-1 inhibitors (org 25935 Schoemaker et al., 2014, AMG747 Dunayevich et al., 2017) failed in their clinical trials. However, these drugs (such as sodium benzoate, D-serine) have potential in clozapine-resistant/treatment-resistance schizophrenia, and dual activation of GMS and antioxidants is viewed as a novel treatment for treatment-resistance schizophrenia (Lin et al., 2020).

**TABLE 2:** Summary of published meta-analyses on NMDAR-targeted drugs.

Year	Drugs	Negative symptoms	Cognitive dysfunction
2010	glycine, D-serine, DCS, sarcosine	SMD = 0.38, 95% CI = 0.19 to 0.56, *p* < 0.0001	SMD = 0.28, 95%CI = 0.10 to 0.47, *p* = 0.002
2011	glycine, D-serine, DCS, sarcosine, NAC,	SMD = -0.27, 95% CI = -0.49 to -0.05, *p* = 0.01	
2013	DCS, D-serine	SMD = 0.62, 95% CI = 0.34 to 0.90, *p* < 0.0001	SMD = 0.06, 95%CI = -0.22 to 0.35, *p* = 0.661
2015	D-serine, DCS, benzoate, NAC		SMD = 0.08, 95%CI = -0.06 to 0.23, *p* = 0.57
2019	D-serine, DCS, benzoate and NAC		SMD = 0.068, 95%CI = -0.056 to 0.193, *p* = 0.283

### The Possible Reasons for Unsatisfactory Clinical Results

These unsatisfactory clinical results challenged the NMDAR hypofunction hypothesis in schizophrenia pathology. There are some conflicting results in this hypothesis. (1) The strongest evidence for this hypothesis is that PCP and ketamine induce all symptoms in schizophrenia. However, the latest studies have revealed that PCP and ketamine do not function exclusively as NMDAR antagonists in the brain (Seillier and Giuffrida, 2009; Zanos and Gould, 2018; Fujigaki et al., 2019; Wang et al., 2020). (2) Early-life NMDAR ablation eliciting schizophrenia-like symptoms were revealed in an animal model (Belforte et al., 2010) but has not been confirmed in humans. The neurodevelopmental differences among species may be overcame *via* models from human induced pluripotent stem cells—a research direction that is worth exploring (Brennand et al., 2011; Noh et al., 2017). (3) Many studies found that NMDAR antagonists can also relieve the symptoms in patients with schizophrenia, which is in contrast to the NMDAR hypofunction hypothesis (Matsuda et al., 2013).

We should however, analyze the pharmacological mechanisms of these drugs in detail before challenging the NMDAR hypofunction hypothesis. In a genetically engineered NMDAR hypofunction model for schizophrenia, NMDAR hypofunction had spatial and temporal boundaries (Nakazawa et al., 2017): (1) The origin of NMDAR hypofunction was reported in cortical and hippocampal GABAergic neurons, particularly in parvalbumin-positive fast-spiking interneurons (Nakazawa and Sapkota, 2020). (2) NMDAR hypofunction happened in the postnatal period, which is before the maturation of parvalbumin-expressing interneurons, and which can cause schizophrenia-like behaviors (Belforte et al., 2010; Lewis et al., 2012; Hardingham and Do, 2016). A new hypothesis named *Dual NMDAR hypofunction* has been brought up by Nakazawa et al. (2017). It suggests that NMDAR hypofunction in cortical and hippocampal GABAergic neurons in early postnatal development serve as a vital starting point, causing cortical circuitry maturation defects, an oxidative stress increase, dopamine dysregulation, etc. The first NMDAR hypofunction elicited the emergence of the three symptom clusters in schizophrenia. A second NMDAR hypofunction in pyramidal neurons then results from the Excitation/Inhibition imbalance and glutamate spillover, which may worsen the negative symptoms and cognitive dysfunction in the progression of schizophrenia (Nakazawa et al., 2017).

First, these NMDAR-targeted drugs may have the imprecise targets for brain regions and cell types. NMDARs are distributed universally in the brain. They are localized mainly in post-synaptic regions with a small percentage being localized in extra-synaptic and pre-synaptic regions (Hardingham and Bading, 2010; Parsons and Raymond, 2014). The impaired NMDARs in schizophrenia pathology were supposed to be synaptic GluN2A-containing NMDARs in cortical and hippocampal GABAergic interneurons (Paoletti et al., 2013). However, these NMDAR-targeted drugs were all systematically administered and few have preference for the cortex and hippocampus. Some drugs such as glycine and GlyT-1 inhibitors failed to augment synaptic NMDARs but target extra-synaptic NMDARs (Papouin et al., 2012). On the contrary, D-serine serves as a synaptic NMDAR co-agonist (Papouin et al., 2012), and endogenous D-serine is co-localized with NMDARs and mainly in GABAergic neurons (Hashimoto et al., 1993). Thus, drugs associated with D-serine elicited stronger efficacy in clinical studies than those associated with glycine. The mGluRs and KATIIs are mainly expressed in GABAergic interneurons in the cortex, which are consistent with the origin of NMDAR hypofunction in schizophrenia (Alexander, 2007; Plitman et al., 2017). Accordingly, the mGluR PAMs and KATII inhibitors elicited good outcomes in pre-clinical studies and have potential to have good results in patients with schizophrenia (Matosin and Newell, 2013; Ellaithy et al., 2015). DAAOs are expressed mainly in cerebellum with little expression in the frontal cortex (Jagannath et al., 2017), so, DAAO inhibitors may not precisely target schizophrenia. GlyT-1s are expressed mainly in glial cells and in glutamatergic neurons rather than GABAergic interneurons (Borowsky et al., 1993), which may account for the failure of many GlyT-1 inhibitors. GABAergic interneurons, especially parvalbumin-positive neurons, have susceptibility to oxidative stress, thus the antioxidants had robust results in reversing the three symptom clusters in patients with schizophrenia (Steullet et al., 2010; Phensy et al., 2017a).

Second, the current treatment may be too late for intervention. NMDAR hypofunction in early-life may be associated with genetic and environmental risk factors. Genetic factors leading to NMDAR hypofunction include rare mutations in NMDAR-encoding genes (Yu et al., 2018) and gene sets associated with NMDAR-mediated synaptic currents (Wang et al., 2018). Environmental risk factors associated with NMDAR hypofunction include stress events or virus infections that occur in early-life or in the prenatal period, which can induce oxidative stress and neuroinflammation in GABAergic interneurons (Nakazawa and Sapkota, 2020). In animal models, NMDAR hypofunction reversed in early-life as NMDAR-targeted drugs can reverse the schizophrenia-like behavioral abnormalities (Phensy et al., 2017a,b). However, in studies, most NMDAR-targeted drugs were administered after the onset of schizophrenia, except for D-serine which showed good efficacy in prodromal symptoms in a clinical high-risk group (Kantrowitz et al., 2015). The effects of early-life administration of NMDAR-targeted drugs in genetic or clinical high-risk groups require further exploration.

## Conclusion

The general effects of NMDAR-targeted drugs were small to moderate in negative symptoms, and small or nonsignificant in cognitive dysfunction in schizophrenia. When comparing the NMDAR-targeted drugs with mainstream antipsychotics, both the NMDAR-targeted drugs and mainstream antipsychotics cannot improve all three symptom clusters of schizophrenia. Can we conclude that the starting point in schizophrenia pathology is neither dopamine disorder nor NMDAR hypofunction? Not yet. However, these two hypotheses interacted with each other. In animal models, GluN1 ablation in GABAergic interneurons at the prenatal period can elicit a hypodopaminergic state in the cortex and a hyperdopaminergic state in the mesolimbic pathway, which is consistent with the dopamine disorder in schizophrenia (Field et al., 2011; Nakazawa et al., 2017). The main dopamine receptor involved in schizophrenia pathology, D2 dopamine receptors (D2Rs), can regulate the NMDAR function and then reverse cognitive dysfunction. In the D2R-expressed pyramidal neuron, the deletion of GSK3β, which took part in the downstream signaling for D2Rs, can upregulate the expression and function of NMDARs (Li et al., 2009, 2020).

Further exploration of the NMDAR hypothesis and therapeutic strategies based on it may bring forward a breakthrough in improving negative symptoms and cognitive dysfunction of schizophrenia. It may provide patients suffering from schizophrenia functional recoveries and normal lives.

## Author Contributions

QW, JH, and RW wrote and revised the review. All authors contributed to the article and approved the submitted version.

## Conflict of Interest

The authors declare that the research was conducted in the absence of any commercial or financial relationships that could be construed as a potential conflict of interest.

## Footnotes

1 http://clinicaltrials.gov/show/NCT01323205

2 http://clinicaltrials.gov/show/NCT00921804

3 http://clinicaltrials.gov/show/NCT00986531

## Abbreviations

MMN: mismatch negativity
GMS: glycine modulatory site
DCS: D-cycloserine
KYNA: kynurenic acid
GlyT-1: glycine transporter-1
PAMs: Positive allosteric modulators
mGluRs: metabotropic glutamate receptors.
